# Automatic Combination of Microfluidic Nanoliter-Scale Droplet Array with High-Speed Capillary Electrophoresis

**DOI:** 10.1038/srep26654

**Published:** 2016-05-27

**Authors:** Q. Li, Y. Zhu, N.-Q. Zhang, Q. Fang

**Affiliations:** 1 Institute of Microanalytical Systems, Department of Chemistry, Zhejiang University, Hangzhou 310058, China; 2 Lishui Center for Disease Control and Prevention, Lishui 323000, China

## Abstract

In this paper, we developed a novel approach for interfacing a microfluidic two-dimensional droplet array to a high-speed capillary electrophoresis (HSCE) system. Picoliter-scale sample injection (ca. 200 pL) from a nanoliter-scale droplet array covered by nonvolatile oil was automatically achieved using the spontaneous injection mode, without the interference from the cover oil and the need of special droplet extraction interface as in previously reported systems. The system was applied in consecutive separations of 25 different samples of amino acids with a whole separation time less than 15 min, as well as on-line monitoring of in-droplet derivatizing reaction of amino acids by fluorescein isothiocyanate (FITC) over 3 hours. High separation speed (up to 100 samples per hour) and high separation efficiency (up to 9.22 × 10^5^ N/m) were achieved.

The past decade has seen increased attention focused on droplet-based microfluidics as an attractive platform for analyzing multiple samples with nanoliter- to picoliter-scale volumes owing to the good isolation and protection effects of immiscible phase to droplets. Various detection techniques including microscopic imaging^1^,^2^, fluorescence and absorption spectrometry^3^, mass spectrometry^4^, capillary electrophoresis (CE)^5^, and liquid chromatography^6^,^7^, have been coupled with droplet-based microfluidic systems. Among them, fluorescence and absorbance detection are the frequently used techniques for analyzing the components in droplet samples, mainly due to the ease of implementation. However, they are difficult to measure samples with complex compositions. The high-resolution separation technique, such as capillary electrophoresis^5^, liquid chromatography^6^,^7^, or mass spectrometry^4^, is capable of performing analysis of complex samples. However, due to the small volumes of droplet samples and the interference from the oil phase used in most droplet systems for compartmenting aqueous droplets, the combination of microfluidic droplet systems with high-resolution separation analysis still presents challenges.

High speed capillary electrophoresis (HSCE), which was first proposed by Jorgenson and Monnig in 1991^8^, is a type of CE technique with fast separation speed and high separation efficiency over traditional CE technique. Usually a typical HSCE system can achieve fast sample separation within dozens of seconds and with separation efficiency up to micrometer or submicrometer plate heights, employing essential conditions including short separation length (*e.g.* <15 cm), narrow injected sample plug (e.g. <100 μm), and high separation electric field strength (*e.g.* >500 V/cm)^8^,^9^,^10^,^11^. Thus, HSCE systems can offer droplet-based microfluidic systems an effective solution for analyzing droplets with complex composition.

In most of the previously reported HSCE systems coupled with droplet-based microfluidic systems, microfabricated chips integrating both continuous-flow droplet module and CE module were frequently adopted^5^,^12^,^13^,^14^,^15^,^16^,^17^,^18^,^19^,^20^. An interface connecting the droplet channel with the CE channel was usually used to transfer droplets from immiscible phase flow to aqueous phase flow. In 2006, Chiu’s group^5^ described a microchip coupling droplet generation with CE separation and laser induced fluorescence (LIF) detection using an interface with a T-junction channel design. A T-junction channel was employed to generate a femtoliter-scale aqueous droplet and then the droplet was transferred to the immiscible boundary of the interface, and fused with (i.e. injected into) the CE buffer flow in the CE channel for subsequent separation and detection. They applied this device in the fast separation of ∼10 fL droplets containing a mixture of fluorescein-labeled amino acids. DeMello’s group^12^ proposed a two-dimensional high performance liquid chromatography (HPLC)-CE system in 2009. The outlet of a capillary HPLC column was connected to a microchip to segment the separated components of a peptide mixture sample by HPLC separation into nanoliter-scale droplets with oil. The formed droplets were delivered into another microchip with an F-shaped channel interface and a passive pillar array to filter out the oil, and then were sequentially extracted from the segmented flow into the continuous CE channel for performing second dimension CE separation. Kennedy’s group^13^,^14^ reported a droplet extraction interface with a K-shaped channel design and a shallow and hydrophilic extraction bridge, which could extract aqueous droplets from the oil stream into the sample loading channel of a CE module with cross channel configuration. HSCE separation could be achieved by using the gated injection method for sample injection, with higher separation efficiencies up to 10^5^ plates/m and short separation time less than 50 s for six amino acids. The device was applied in the *in-vivo* monitoring of rapid concentration changes evoked by infusing glutamate uptake inhibitor into the striatum of anesthetized rats. In 2010, the same group^15^ presented a system integrating three parallel K-shaped droplet extraction interfaces and electrophoresis elements on one microchip to further increase analysis throughput to 120 samples/10 min. In 2015, Hassan *et al*. used the Slipchip technique to achieve the injection and gel electrophoresis separation of multiple 30 sub-nL sample droplets^20^. Thirty nanoliter-sacle droplets of dyes or DNA fragments were first formed in parallel by slipping the chip and then moved to be in contact with the separation channels for CE separation.

In contrast to continuous-flow droplet systems formed in microchip channels^5^,^12^,^13^,^14^,^15^,^16^,^17^,^18^,^19^, two-dimensional droplet array systems built on microwells or microregions on microchips, especially those with open or semi-open features^21^,^22^,^23^,^24^,^25^,^26^, have more practical benefits of easily handling massive different sample droplets with a capillary probe. In 1998, Whitesides’ group^21^ reported a simple method to form high-density droplet array using the spreading and discontinuous dewetting of a sample solution on a microwell array chip. A CE system was also preliminarily coupled to an open droplet system formed by this method for performing enzyme assay. The capillary was directly inserted into the droplet without cover oil for performing reagent addition and sample introduction. The reactants and product were separated within 100 s with an effective separation length of 15 cm. For an open droplet array system with nanoliter-scale droplet volumes, usually a nonvolatile oil is required to cover the droplets to avoid droplet evaporation. However, the use of cover oil will interfere with the sampling from droplets and disturb the subsequent CE analysis^27^. To address this problem, Litborn *et al*.^27^. described a method to suppress the evaporation of droplets on a silicon microvial array chip (15 nL volume for each vial) using a thin, flowing film of a volatile liquid (e.g., octane) to cover the droplet array. In-droplet myoglobin digestion reaction and CE separation for the enzymatic products in droplet were performed. A sample plug of 5 nL droplet solution was injected into the capillary for CE separation by inserting the capillary inlet through the octane into the droplet, and applying vacuum to the outlet end of the capillary, without the interference from the octane cover. The peptides after digestion in droplet were separated with an effective separation length of 60 cm in 18 min. However, in spite of the above significant progress in microfluidic droplet-CE techniques, there are still challenges in developing sample introduction interfaces between droplet-based microfluidics and high speed CE capable of performing picoliter-scale sample injection and fast separation of large number of different samples, which are in urgent needed in high-throughput screening and analysis.

In the present study, we developed a novel approach for interfacing microfluidic droplet array with HSCE using the spontaneous sample injection technique^10^ to achieve picoliter-scale injection without the need of droplet extraction interface. Based on this approach, we built an automated nanoliter droplet array-HSCE system (Fig. 1) for analysis of multiple different samples. The system was applied in the automated and continuous separation of a series of droplet samples of fluorescein isothiocyanate (FITC)-labeled amino acids with laser-induced fluorescence detection, showing comparable separation speed and efficiency to those of previous HSCE systems based on microchips or short capillaries.

## Results

### Picoliter-scale sampling from droplet array

In the previously reported microfluidic droplet-CE systems with aqueous droplet phase and immiscible, nonvolatile oil phase for the compartmentation of droplets^5^,^12^,^13^,^14^,^15^,^16^,^17^,^18^,^19^, usually microchip-based continuous flow mode in microchannel network was employed. In these systems, various types of droplet extraction interfaces were commonly required to firstly extract aqueous droplets from segmented flow into aqueous phase channel, and then to inject the aqueous droplet samples into channel for CE separation sequentially. In this work, we used a simple method to achieve picoliter-scale sample injection from droplet array without the need of droplet extraction interface. The separation capillary was also used as a sampling probe, and directly inserted into the target droplet through the cover oil to perform spontaneous injection based on surface tension. The key factor to the success of such a sampling strategy lies in how to avoid the interfering of the cover oil in the sampling process. In the early stage of this work, on the basis of our previous sampling methods for droplet^23^,^26^ and HSCE^10^ systems, we made hydrophobic modification to the outer surface of the capillary probe tip end with a salinizing agent of dimethyldichlorosilane to reduce carryover in sampling for multiple samples. We used a 1.0 × 10^−2^ M fluorescein solution as a model sample for the observation of the sample introduction process by a stereo microscope. However, with such a method, no obvious sample injection phenomenon was observed during the spontaneous injection process. To further understand the spontaneous injection process of capillary from oil-covered droplets, we used a high-speed CCD camera (Phantom micro 3, Vision research, Wayne, USA) equipped with a microscope lens (Zoom 6000, Navitar, Rochester, USA) to record the sample injection process with 4000 fps by first inserting the tip end of a capillary filled with borate buffer into an oil-covered droplet of 1.0 × 10^−2^ M fluorescein solution, and then allowing the droplet to perpendicularly remove from the capillary tip by the translational stage. A series of images captured from Video S1 (see Supporting Information) recording the injection process are as shown in Fig. 2a. With the removing of the droplet from the capillary tip, the liquid bridge between the capillary tip and the droplet tended to be thinner (Fig. 2a3–a5). When the liquid bridge finally broke up, few sample solution could be observed to be remained at the capillary tip (Fig. 2a6), due to the lipophilic property of the outer surface of the capillary tip and the scraping function of the oil phase to the capillary tip during the removing process. Thus, there was no evident sample injection could be obtained in CE experiment with such a capillary.

**Figure 1:**
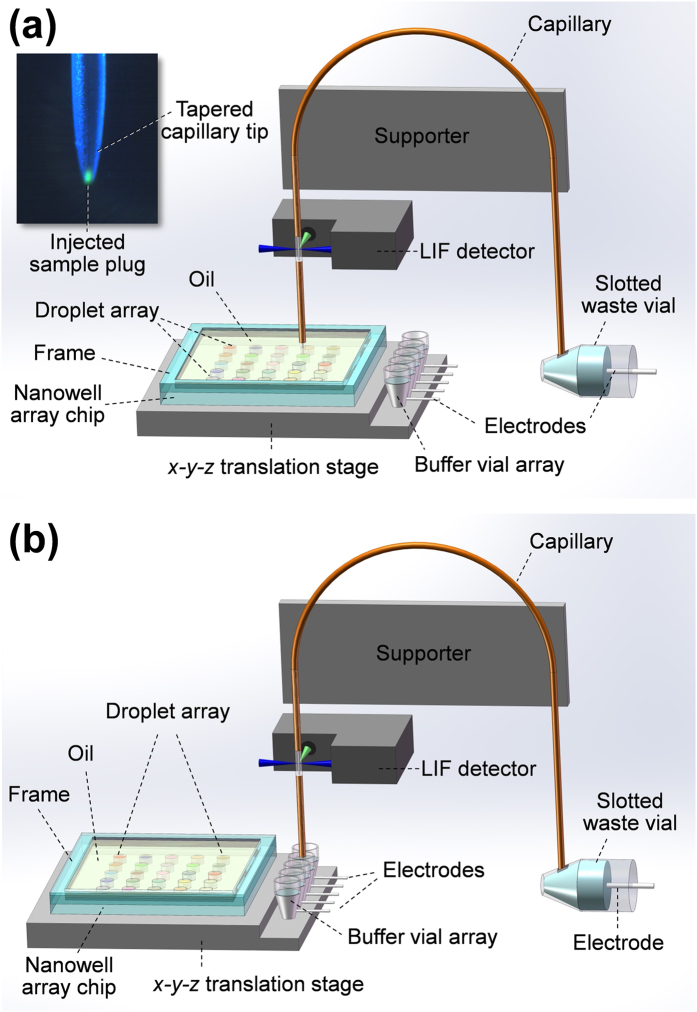
Schematic diagram of the HSCE system coupled to a droplet array. (**a**) Sample injection stage with the capillary tip end inserted into the sample droplet. The inset shows an image of the capillary tip end after a spontaneous injection of a model sample of 1 × 10^−3^ M sodium fluorescein solution. (**b**) Sample separation stage with the capillary tip end inserted into buffer vial for performing the CE separation of the injected sample plug.

**Figure 2:**
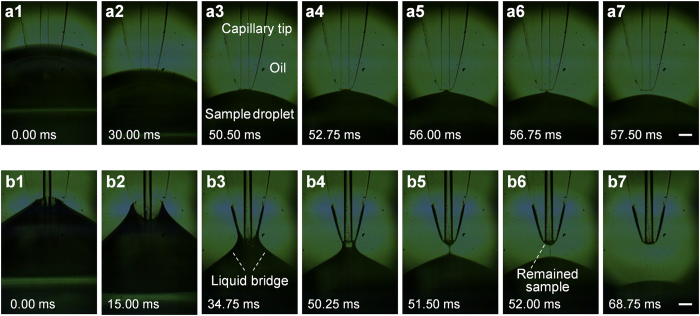
Series of images captured by a high-speed CCD camera in the spontaneous injection process of a capillary probe from a droplet sample. (**a**) Capillary probe with hydrophobic outer surface on the tip end; (**b**) Capillary probe with hydrophilic outer surface on the tip end. Conditions: tapered-tip capillary, 50 μm i.d.; sample droplet, 1.0 × 10^−2^ M fluorescein solution; capillary tip removing speed, 1000 mm/min.

We further tried to use a capillary without surface modification, i.e. with hydrophilic inner and outer surfaces, to perform sample injection. As shown in Fig. 2b (see Video S2 in Supporting Information), the pattern and variation of the liquid bridge between capillary tip and the droplet of the hydrophilic capillary (Fig. 2b3–b5) were quite different from those of the hydrophobic capillary. Due to the hydrophilic property of the outer surface of the capillary tip, more droplet sample solution was adhered to the capillary tip sidewall during the droplet removing process, and thus more sample solution remained at the capillary tip end when the liquid bridge broke up (Fig. 2b6). The remained sample at the capillary tip was rapidly sucked into the capillary channel by surface tension (Fig. 2b7) to achieve the spontaneous sample injection. Observed with a fluorescence microscope (Fig. 1a), picoliter-scale injection ca. 200 pL (100 μm plug length) could be achieved under the in-oil spontaneous injection mode. Such an injection volume is similar to those in homogeneous solution systems^10^,^28^, which ensure the high separation efficiency in the subsequent CE separation. During the in-oil sample injection process, even in continuous injections for multiple samples, no oil was aspirated into the capillary channel, and no interference effect from the cover oil to the CE separation processes was observed.

Actually, for spontaneous injection of homogeneous sample solutions, the use of unmodified capillary would lead to relatively large cross-contamination between different samples due to the residual of sample solution on the sidewall of the capillary tip end^13^. We tested the cross-contamination of the present system by continuously performing 200 injections and separations of a concentrated fluorescein sample (1.0 × 10^−3^ M). We further measured the concentration of fluorescein in the borate buffer solution after 200 separations, which was carried by the capillary tip when it repetitively switched between the sample droplet and buffer solution vial. A cross-contamination from the sample of ca. 0.14% was obtained, which could be omitted. This significantly low carryover could be attributed to the washing and brushing effect of the cover oil to the sidewall of the capillary tip end when the capillary removed from the sample droplet and through the cover oil during the sample injection process.

### Optimization of the system

We investigated various factors affecting the spontaneous injection process, including the type and volume of cover oil, aqueous sample droplet volume, and removing speed of the capillary tip.

Three oils frequently used in droplet-based microfluidic systems, silicone oil, tetradecane and fluorinated oil (Fluorinert ^®^FC-40, Sigma-Aldrich)^29^, were tested as the cover oil, and their performance in the separation of a mixture of three amino acids were compared with the same sample without cover oil. The results showed no evident difference in peak heights and separation efficiencies between the experiments with different oils as well as without oil. Silicone oil was chosen as the cover oil due to its good nonvolatility, as well as chemical and biological compatibility. We also tested the effect of cover oil volume, i.e. the thickness of the cover oil layer, on the spontaneous injection process, with different oil volumes of 100, 200, 300 and 400 μL, corresponding to oil layer thickness of 440, 890, 1330, and 1780 μm. There is no evident variation (<6%) in injection volume estimated from the peak areas of amino acids. A 200 μL of silicon oil could effectively avoid droplet evaporation during the analysis process.

We also tested the effects of droplet volume using droplets with volumes of 100, 200, 300, 400, and 500 nL. When the droplet volume was larger than 300 nL, the spontaneous injection could be achieved without evident effect of the droplet volume on the injection process. When the droplet volume was smaller than 200 nL, the spontaneous injection was unable to be conducted normally (as shown in Figure S1). This was because the 200-nL droplet exhibited a concave meniscus shape in the 1-mm-diameter well and had a droplet height lower than ca. 250 μm, under which condition the x-y-z translation stage used in the present work could not accurately control the movement of the droplet array to allow the capillary tip insert into the droplet. If required, further reducing the droplet volume to the range of several tens nanoliters to several hundred picoliters could be achieved by using microwells with smaller diameters, and using x-y-z translation stage with higher moving precision to ca. 10 μm, as in previously reported SODA system^26^.

As reported previously, the removing speed of capillary tip end from sample solution has significant effect on the spontaneous injection volume^10^. We studied the effects of the removing speed of the capillary tip end from sample droplet on the spontaneous injection in the removing speed range of 30–1000 mm/min (Figure S2). In the removing speed range of 50–1000 mm/min, the sample plug volume reflected by the peak area, shows a slight decreasing trend with the increase of the removing speed, and correspondingly the separation efficiency (plate number) shows an increasing trend with the removing speed and reaches the highest level at 1000 mm/min. Therefore, the removing speed of 1000 mm/min that was the highest one available in the present system was chosen to increase the separation efficiency and analysis throughput.

### Performance of the system

**Figure 3:**
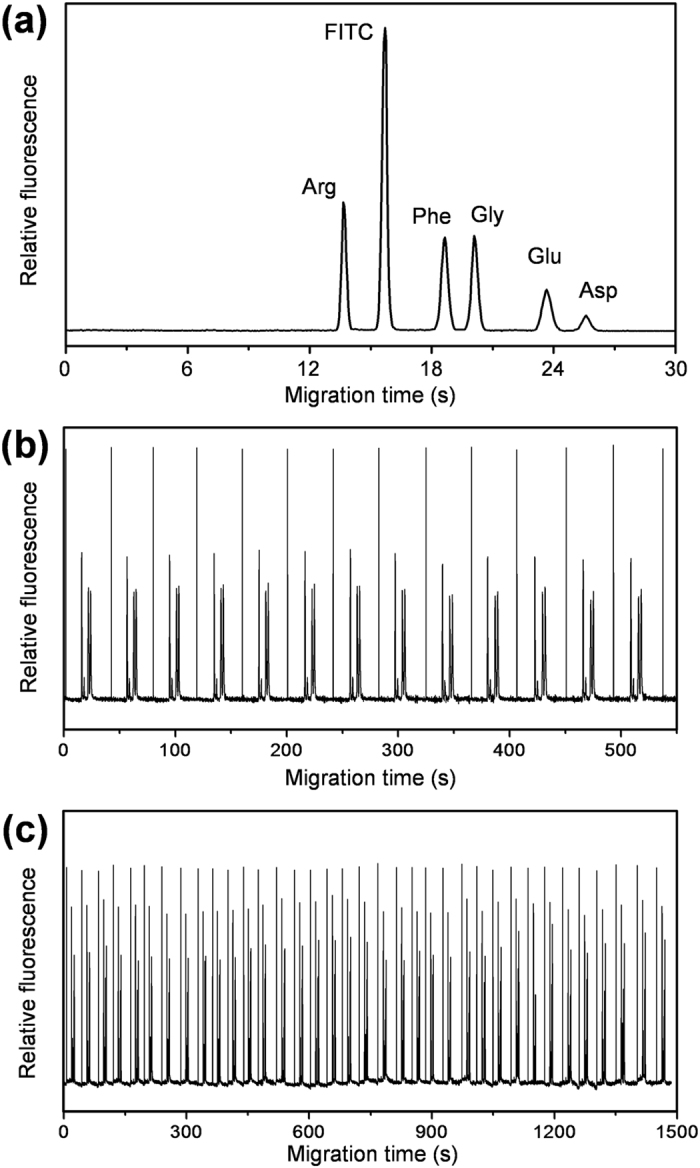
(**a**) A typical electropherogram of a mixture of five FITC-labeled amino acids. (**b**) Electropherogram of continuous separation of the same sample droplet of a mixture of FITC-labeled amino acids in repeatability experiment. (**c**) Electropherogram of the repeatability experiment for continuous separation of 25 sample droplets in different nanowells of the droplet array with the same composition of a mixture of three FITC-labeled amino acids. Conditions: tapered-tip capillary, 50 μm i.d.; effective separation length, 2.0 cm; oil volume, 200 μL; capillary removing speed, 1000 mm/min; separation field strength, 350 V/cm; working electrolyte, 5 × 10^−3^ M borate buffer (pH 9.2).

Under the optimized conditions, the present system was first applied in the separation of droplet sample of a mixture of FITC-labeled amino acids. As shown in Fig. 3a, five FITC-labeled amino acids including arginine, phenylalanine, glycine, glutamic and asparagine are separated in 30 s with a 2.0-cm long effective separation length and 350 V/cm electric field strength (Figure S3). High separation efficiencies ranging from 5.86 × 10^5^/m to 9.22 × 10^5^/m (corresponding to 1.71 μm to 1.08 μm plate heights) are obtained for the amino acids, which are comparable to most of the microchip-based HSCE systems.

Figure 3b shows the electropherograms obtained in a repeatability experiment for a droplet sample of a mixture of three FITC-labeled amino acids. The repeatabilities for the peak height, and migration time of these FITC-labeled amino acids are in the range of 2.6–3.1% and 2.1–2.7% relative standard deviation (RSD), respectively (n = 13). We also tested the repeatability of the present system in automated separation of multiple sample droplets in different nanowells by first generating a sample droplet array of 25 droplets with the same composition of a mixture of three FITC-labeled amino acids, and then sequentially sampling and separating these droplet samples within 25 min (Fig. 3c). The RSDs of peak height and migration time of the 25 droplets were in the range of 3.2–4.4% and 3.0–4.4%, respectively (n = 25). These results demonstrate a good consistency for the droplets in different nanowells in the droplet array and high working stability of the present system.

**Figure 4:**
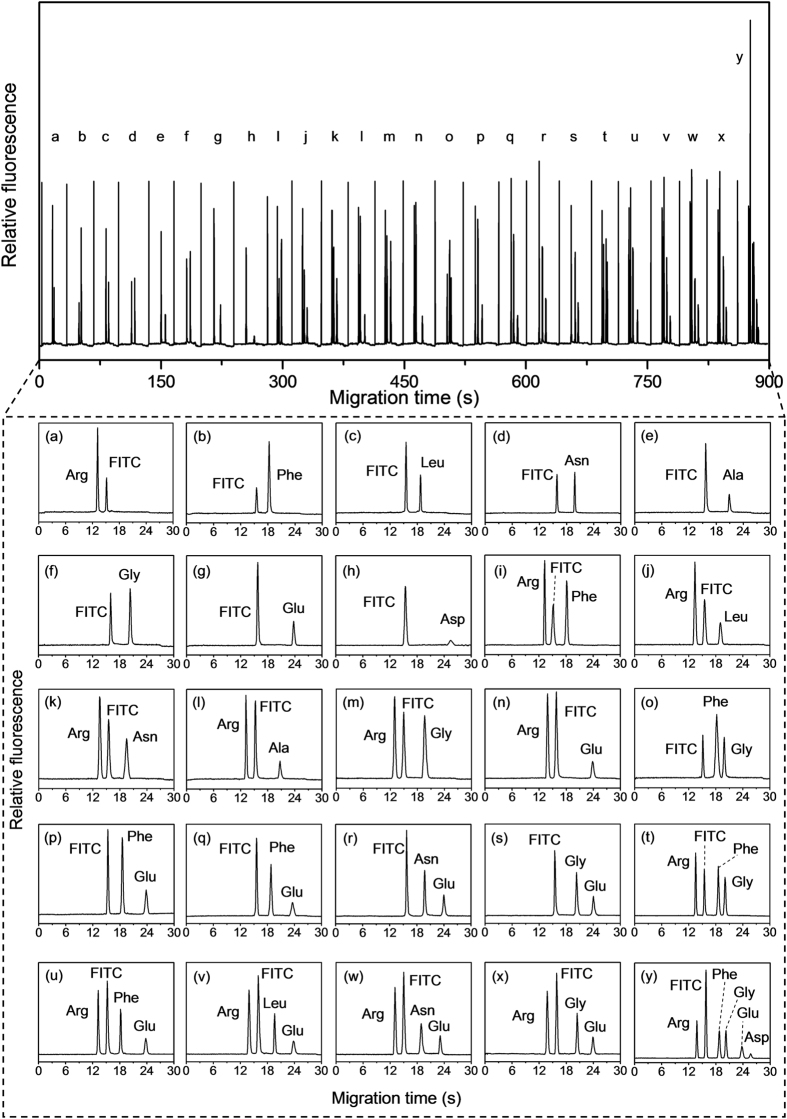
Electropherograms of consecutive separations of 25 different sample droplets of FITC-labeled amino acid mixtures. Conditions as in Fig. 3.

The present system was also applied in the consecutive separations of 25 different droplet samples of FITC-labeled amino acids (Fig. 4). All of the analytes in the 25 different droplet samples were baseline resolved, and the whole separation was achieved in less than 15 min.

**Figure 5:**
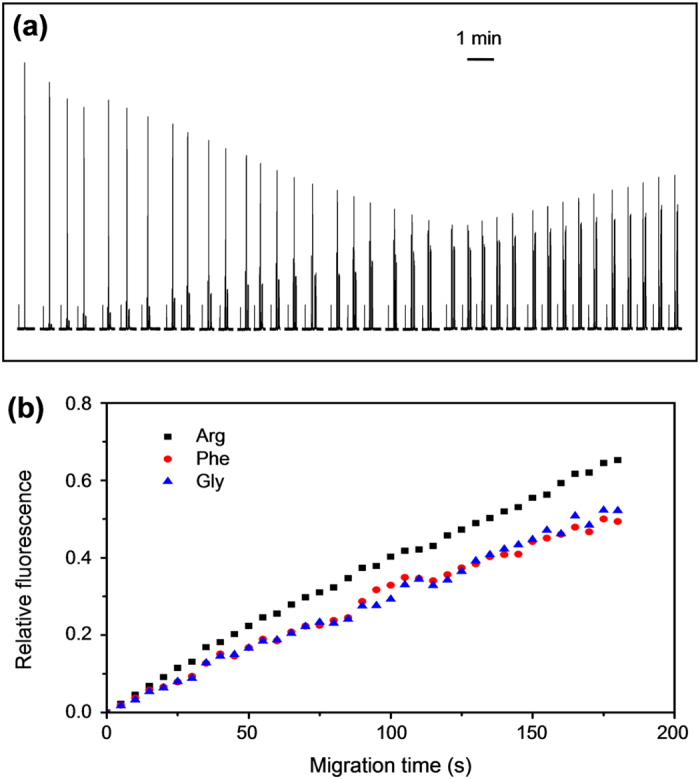
(**a**) Electropherograms of on-line monitoring of in-droplet derivatizing reaction of amino acids. Conditions: reaction temperature. 28 °C; concentration of arginine, FITC, phenylalanine and glycine, 10^−4^ M; CE conditions as in Fig. 3. (**b**) The relative fluorescence *vs* time curve of arginine, phenylalanine and glycine in (**a**).

Moreover, we also applied the present system in the on-line monitoring of in-droplet derivatizing reaction of amino acids. FITC and three amino acids including arginine, phenylalanine, and glycine were added in a 500 nL sample droplet, and then sampling from the droplet and CE separation were carried out every 5 min with a whole monitoring time of 3 h (Fig. 5a,b). The results demonstrate that this system has the ability of performing long-term on-line monitoring to nanoliter droplet reactors.

## Conclusion

In summary, we have developed an automated microfluidic droplet array-HSCE system using a simple interfacing approach to couple the two systems. The combined system has simple structure and is easy-to-build without the need of microfabricated CE chip and droplet extraction interface. It is capable of performing automated high-speed and high-efficiency CE separation for multiple different nanoliter-scale droplet samples. Compared with the droplet systems under continuous flow mode, the two-dimensional droplet array system has the advantages of large capacity for containing large number of sample droplets, as well as easily addressing and handling each droplet in the array by the droplet position information, such as repeatedly sampling from one droplet for on-line monitoring in-droplet reaction (Fig. 5). During the processes of continuous sample analysis and long-term on-line monitoring, the present system exhibited high degree of automation and working reliability.

This work provides a novel strategy for building an automated and versatile platform achieving the combination of droplet-based microfluidics and HSCE. The present system could be further developed into an analysis platform coupled with multiple detectors including fluorescence, absorbance, mass spectrometry and electrochemistry. This will not only significantly expand its application scope, but also offer effective tools for fast analysis and screening of minute amounts of samples, such as in drug discovery, enzyme reaction kinetics research, single cell analysis, and PCR product identification.

## Methods

### Setup of the droplet array-HSCE system

The system mainly consisted of two modules, a microfluidic droplet array and a HSCE module, as shown in Fig. 1. The droplet array module was composed of a nanowell array chip, a buffer vial array cut from a commercial 384-well plate (Axygen Scientific, Union City, USA), and a x-y-z translation stage (Model 3040C, 50 μm moving precision, Dongda Co., Jiangyin, China). The nanowell array chip was fabricated from a chromium glass plate (Shaoguang Microelectronics Co., Changsha, China) using drilling method, to form a 5 × 5 nanowell array (1.0 mm in diameter, 0.5 mm in depth for each well) with a distance of 2 mm between the centers of two adjacent nanowells. The chromium layer on the glass chip surface around the nanowells was preserved, and no special treatment was made to the surface of the chip and nanowells. The natural hydrophobic property of the chromium layer ensured its good compatibility with the cover oil layer for avoiding aqueous droplet evaporation. The hydrophilic property of the nanowell surface made the nanowells to be compatible with aqueous sample droplets. A glass frame with a square bore size of 15 × 15 mm fabricated by grinding a glass plate, was fixed on the top of the nanowell array chip with glue for containing cover oil layer. The nanowell array chip and the buffer vial array were fixed on the x-y-z translation stage and positioned at the same level.

In the HSCE module, a 20 cm-long fused silica capillary (50 μm i.d., 375 μm o.d., polyimide coating thickness 20 μm, Refine Chromatography Co., Yongnian, China) was used as separation column. The inlet end of the capillary was mechanically ground into a tapered tip as described previously^28^. Unless mentioned otherwise, an effective separation length of 2.0 cm was used in the CE separation. The polyimide coating at the detection point of the capillary was removed carefully with a length of ca. 5 mm. The capillary was bent into a “∩” shape, and fixed vertically on a supporter with the inlet and outlet ends of the capillary at the same level. During CE separation process, the inlet end of the capillary was inserted into working electrolyte solution filled in a buffer vial (Fig. 1b). the outlet end of the capillary was inserted into working electrolyte filled in a horizontally placed waste vial through its slot (Fig. 1b). The slotted waste vial was produced from a 0.2-mL PCR tube (Porex, Petaluma, USA) with a 1.0-mm-wide, 2.0-mm-deep slot cut by a razor blade on the tube bottom^23^. A high-voltage power supply, variable in the range of 0 to 30,000 V, was used for the CE separation. The output-grounding terminal and the output-positive high voltage terminal were connected to platinum electrodes in waste vial and buffer vial, respectively.

The analytes were detected by a homebuilt orthogonal laser-induced fluorescence system (λ_ex_ = 405 nm, λ_em_ = 525 nm), which was built as described previously^23^,^30^. Briefly, the laser beam of a 405 nm laser diode was filtered with a 405 nm band-pass filter (HB Optical technology Co., Shenyang, China) and focused on the capillary channel by an objective (40×, Chongqing MIC Co., Chongqing, China). The excited fluorescence was collected by another objective (Xian Xike Co. Xian, China) placed at the plane perpendicular to the laser beam with a detection angle of 45° to the capillary, filtered by a 525 nm band-pass filter (HB Optical technology Co., Shenyang, China), and detected by a photomultiplier (CR114, Hamamatsu photonics, Hamamatsu, Japan).

### Procedures

Before use, the capillary in the HSCE module was rinsed sequentially with 1 M NaOH, deionized water and 5 mM borate buffer. The buffer vials and waste vial were filled with 5 mM borate buffer, with the liquid levels in the two vials on the same level to avoid generating hydraulic pressure in the capillary. The capillary was filled with 5 mM borate buffer with its inlet end immersed in the buffer vial and outlet end in the waste vial. The droplet array of different samples was generated using the sequential operation droplet array (SODA) method as described previously^26^. Spontaneous sample injection and CE separation were performed by moving the x-y-z translation stage, allowing the capillary inlet end first to insert into the sample droplet through the cover oil layer without high voltage applied between the sample droplet and waste vial, and then quickly remove from the sample droplet and the cover oil layer (Fig. 1a). During the removing process of the capillary inlet end from the sample droplet, a pL-scale sample solution remained at the capillary tip end and subsequently was quickly sucked into the capillary channel by surface tension, forming a sample plug (Figs 1a and 2b). Then the capillary tip end was inserted into the buffer vial by moving the x-y-z translation stage, and a high voltage was applied between the buffer and waste vials to carry out CE separation as soon as the capillary tip end was immersed into the buffer solution filled in the buffer vial (Fig. 1b).

## Additional Information

**How to cite this article**: Li, Q. *et al*. Automatic Combination of Microfluidic Nanoliter-Scale Droplet Array with High-Speed Capillary Electrophoresis. *Sci. Rep.* **6**, 26654; doi: 10.1038/srep26654 (2016).

## Supplementary Material

Supplementary Information

Supplementary Information

Supplementary Information
