# Supplementary material for: Automatic Combination of Microfluidic Nanoliter-Scale Droplet Array with High-Speed Capillary Electrophoresis
Source: Sci Rep. 2016 May 27;6:26654. doi: 10.1038/srep26654 (PMC4882528; doi:10.1038/srep26654)
Supplement: Supplementary Information [file srep26654-s3.doc]

**Supporting Information**

**Automatic Combination of Microfluidic Nanoliter-Scale Droplet Array with High-Speed Capillary Electrophoresis System**

Qi Li,^a,b^ Ying Zhu,^a^ Nai-Qian Zhang,**^a^** Qun Fang*^a^

^a^ Institute of Microanalytical Systems, Department of Chemistry, Zhejiang University, Hangzhou310058, China

^b^ Lishui Center for Disease Control and Prevention, Lishui323000, China

Corresponding Author

*E-mail: fangqun@zju.edu.cn. Tel.: +86-571-88206771. Fax: +86-571-88273572.

**Chemicals and Reagents**

All reagents were of analytical reagent grade unless mentioned otherwise, and deionized water was used throughout. Working electrolyte for CE separation was 5 mM borate buffer (pH 9.2). Stock solutions of 1 mM fluorescein isothiocyanate (FITC, isomer I, Sigma Chemicals, St. Louis, USA) labeled amino acids (Kangda Amino Acid Works, Shanghai, China) were prepared as previously described elsewhere.^S1^ The sample solution containing a mixture of 1 μM each amino acid was prepared by mixing the labeled amino acid solutions and diluting with 5 mM borate buffer. A solution of silicon oil and 15% Acid Red 18 (Sinopharm Chemical Reagent Co., Shanghai, China) was used as the oil phase and a model sample for the observation of the sample introduction process, respectively.

**Effects of droplet volume to sample injection.**

We tested the effects of droplet volume using droplets with volumes of 100 nL, 200 nL, 300 nL, 400 nL, and 500 nL. When the droplet volume was larger than 300 nL, the spontaneous injection could be achieved without evident effect of the droplet volume to the injection process. When the droplet volume was lower than 200 nL, the spontaneous injection was not able to be conducted normally (as shown in Figure S3). This was because the 200-nL droplet exhibited a concave meniscus shape in the 1-mm-diameter well and had a droplet height lower than ca. 250 μm, at which condition the *x-y-z* translation stage used in the present work could not accurately control the movement of the droplet array to allow the capillary tip insert into the droplet. If required, further reducing the droplet volume could be achieved by fabricating deep wells with smaller diameter to increase the droplet height, and using *x-y-z* translation stage with higher moving precision.

Figure S1. Electropherograms of a mixture of FITC-labeled amino acids at droplet volumes of 200 nL and 300 nL. Conditions: tapered-tip capillary, 50-µm i.d.; effective separation length, 2.0 cm; removing speed of capillary tip, 1000 mm/min; oil volume, 200 μL; separation field strength, 350 V/cm; working electrolyte, 5 mM borate buffer (pH 9.2).

**Effects of capillary removing speed to sample injection.**

As reported previously,^S1^ the removing speed of capillary tip end from sample solution has significant effect on the spontaneous injection volume.^S2^ We studied the effects of the removing speed of the capillary tip end from sample droplet on the spontaneous injection in the moving speed range of 30-1000 mm/min (as shown in Figure S3). In the removing speed range of 50-1000 mm/min, the sample plug volume, reflected by the peak area, shows a slight decreasing trend with the increase of the removing speed, and correspondingly the separation efficiency (plate number) shows a increasing trend with the removing speed and reached the highest level at 1000 mm/min. Therefore, the removing speed of 1000 mm/min which was the highest one available in the present system was chosen to increase the separation efficiency and analysis throughput.

Figure S2. (a) Electropherograms of a mixture of FITC-labeled amino acids at different removing speeds of capillary tip. (b) The effects of capillary removing speed on separation efficiency of arginine, FITC, phenylalanine and glycin in (a). Conditions are as shown in Figure S1.

**Effects of electric field strength to CE separation.**

We studied the effects of the electric field strength for CE separation. The results are shown in Figure S4. The current *vs*. electric field strength curve of the capillary channel showed a linear relationship between 50 and 350 V/cm and deviated from the linear behavior above 400 V/cm. The migration time decreased with the increase of electric field strength in the range of 50-450 V/cm. The plate numbers of arginine, phenylalanine and glycin increased with the field strength from 100 to 400 V/cm, reached a maximum value at 350 V/cm, and decreased at further higher field strengths due to the zone dispersion caused by the Joule heating effect. In this experiment, 350 V/cm of electric field strength was adopted owing to its high separation efficiency and speed.

Figure S3. (a) Electropherograms of a mixture of FITC-labeled amino acids at different electric field strengths. (b) The effects of electric field strength on separation efficiency of arginine, FITC, phenylalanine and glycine. Conditions as in Figure S1.

**REFERENCES**

[S1] Li, Q., Zhang, T., Zhu, Y., Cheng, Y.Q., Lin, Q.H., Fang, Q. Automated high-speed CE system for multiple samples. *Electrophoresis*. ***34***, 557-561 (2013).

[S2] Zhang, T., Fang, Q., Du, W.B., Fu, J. L. Microfluidic picoliter-scale translational spontaneous sample introduction for high-speed capillary electrophoresis. *Anal. Chem.* ***81***, 3693-3698 (2009).


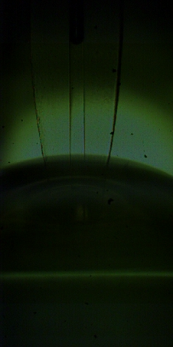


Video S1-Spontaneous injection process of capillary with hydrophobic tip outer surface

A video recording the spontaneous injection process of a capillary probe with hydrophobic tip outer surface from a droplet sample is provided. The video was recorded with a speed of 4000 fps, and it is played at a speed of 10 fps. Conditions: tapered-tip capillary, 50 µm i.d.; sample droplet, 1.0×10^-2^ M fluorescein solution.


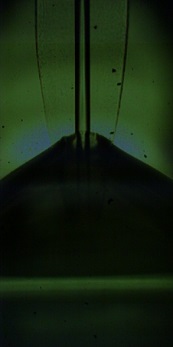


Video S2-Spontaneous injection process of capillary with hydrophilic tip outer surface

A video recording the spontaneous injection process of a capillary probe with hydrophilic tip outer surface from a droplet sample is provided. The video was recorded with a speed of 4000 fps, and it is played at a speed of 10 fps. Conditions: tapered-tip capillary, 50 µm i.d.; sample droplet, 1.0×10^-2^ M fluorescein solution.
